# Supplementary material for: ARMMs as a versatile platform for intracellular delivery of macromolecules
Source: Nat Commun. 2018 Mar 6;9:960. doi: 10.1038/s41467-018-03390-x (PMC5840177; doi:10.1038/s41467-018-03390-x)

Supplementary Information

ARMMs as a versatile platform for intracellular delivery of macromolecules

Wang et al.

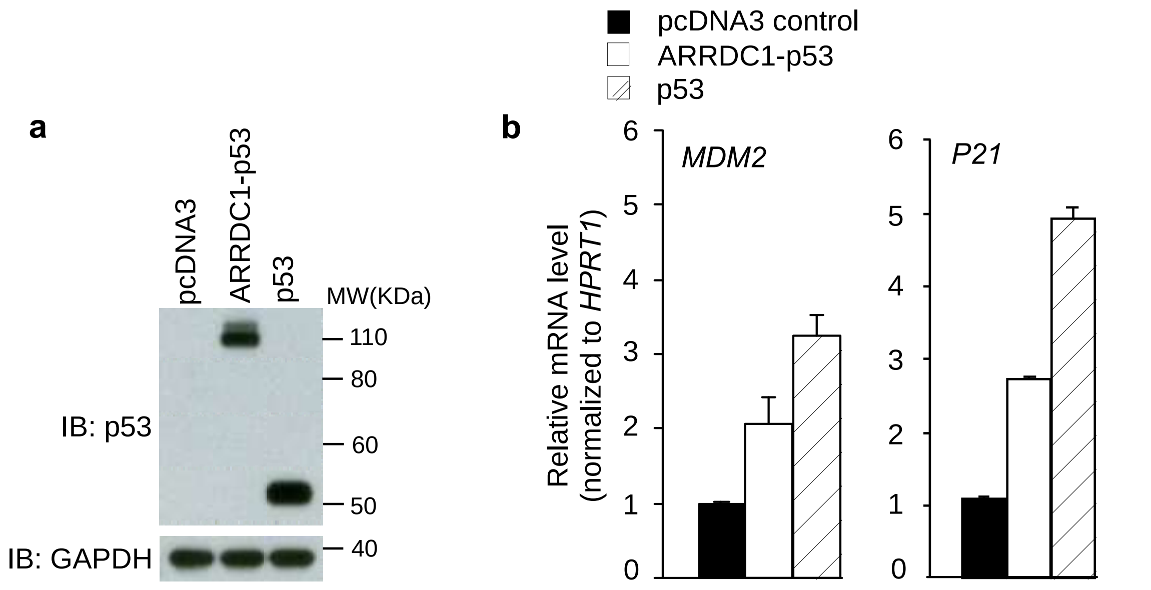

Supplementary Figure 1: Expression and transcriptional activity of ARRDC1-p53 fusion protein. (**a**) Western blotting showing expression of ARRDC1-p53 fusion protein. H1299 cells were transfected with control pcDNA3 vector, ARRDC1-p53 or p53 expression constructs. Cell lysates were used for p53 and GAPDH Western blotting. (**b**) ARRDC1-p53 increased the expression of p53 target genes. H1299 cells were transfected with control pcDNA3 vector, ARRDC1-p53 or p53 expression constructs. Total RNAs were extracted from cells 16 h post transfection. Quantitative RT-PCR was done to measure the expression of MDM2 and p21 mRNAs.

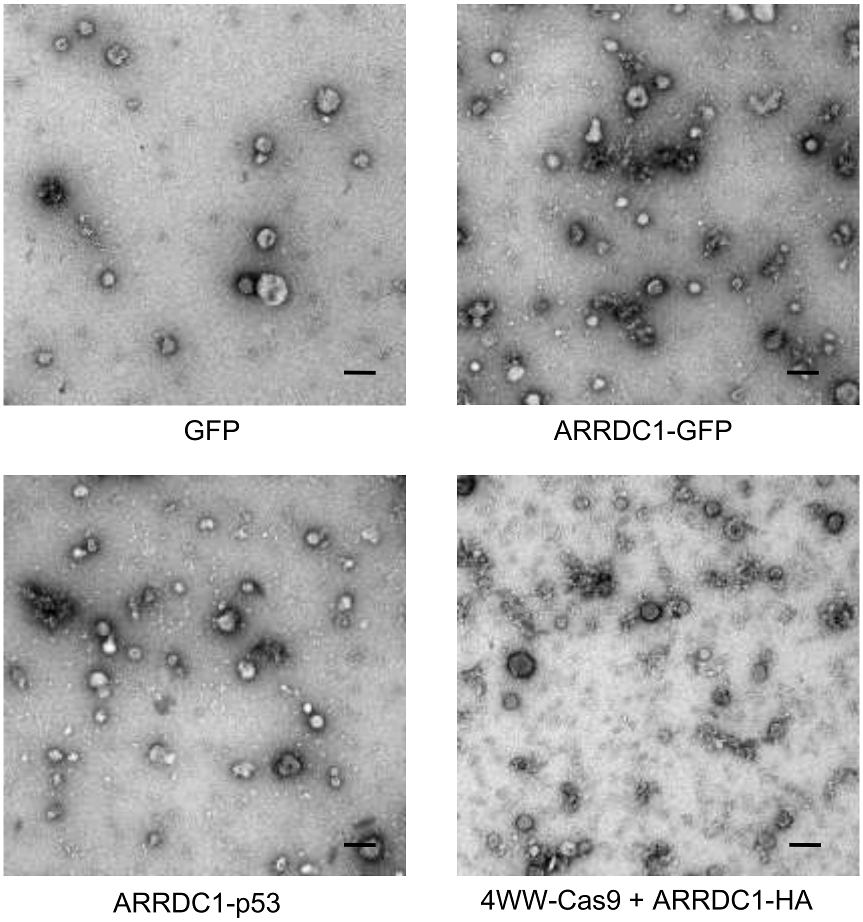

Supplementary Figure 2: Transmission electron microscope (TEM) images of EVs. HEK293T cells were transfected with the indicated expression constructs. EVs collected and purified through sucrose gradient purification were fixed onto carbon coated grid and the images were recorded by JEOL 1200EX transmission electron microscope. The black bar in each figure indicates the length of 100 nm.

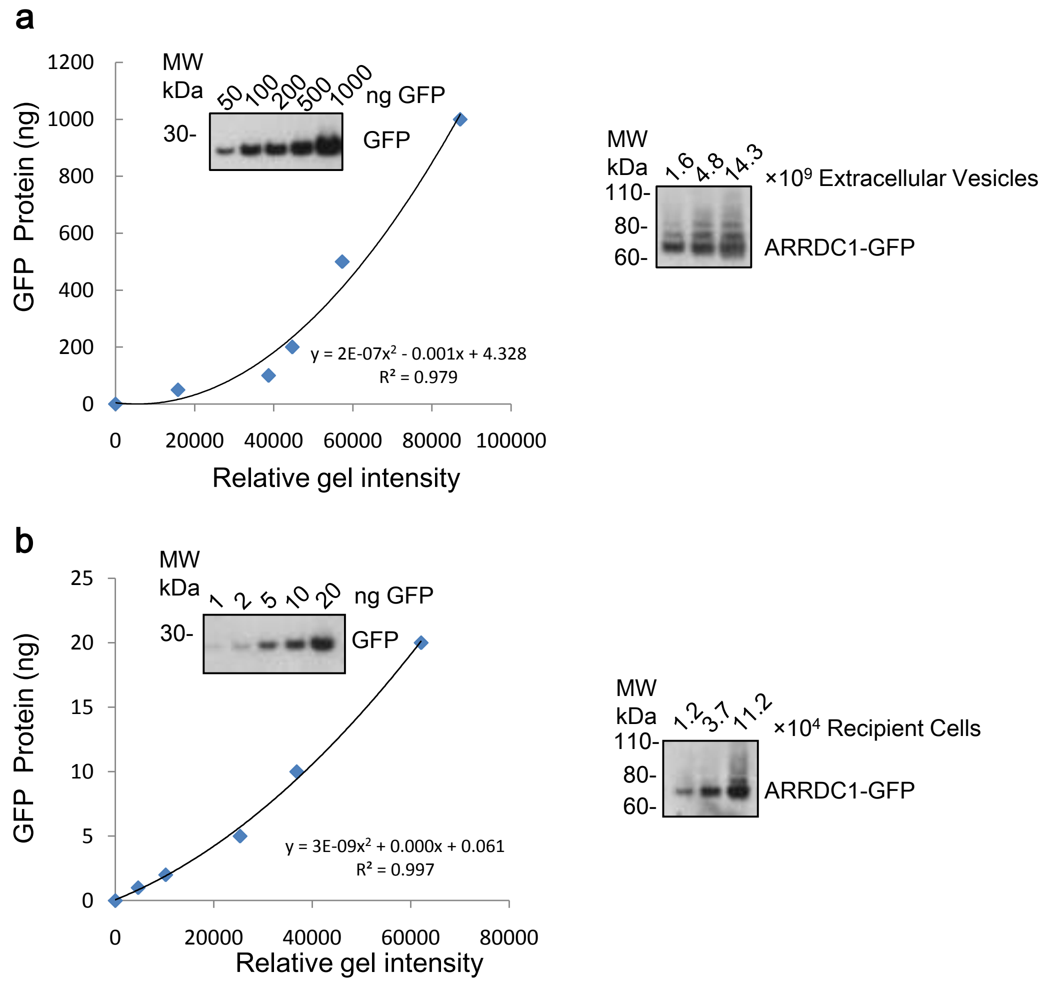

Supplementary Figure 3: Quantification of ARMMs packaging and transfer. Anti-GFP Western blotting was done to quantify the amount of ARRDC1-GFP protein in ARMMs (**a**) and in the recipient cells (**b**) that received ARRDC1-GFP containing ARMMs. Recombinant GFP protein (Novus, NBC1-22949) of indicated amount was used as standards. Samples of ARMMs or lysates of recipient cells were run on the same blots as the corresponding GFP standards. Western blotting films were quantified by ImageJ (Analyze/Gels function) and analyzed in Excel (Trendline options/Polynomial at order 2). We used the mid-range samples of EVs or recipient cells to quantify the GFP protein amount. Our calculations indicated that each ARMM vesicle contains an average of ~540 cargo protein molecules (ARRDC1-GFP) and that each recipient cell received around 3.1×10^6 cargo proteins (and 5.8×10^3 ARMMs).

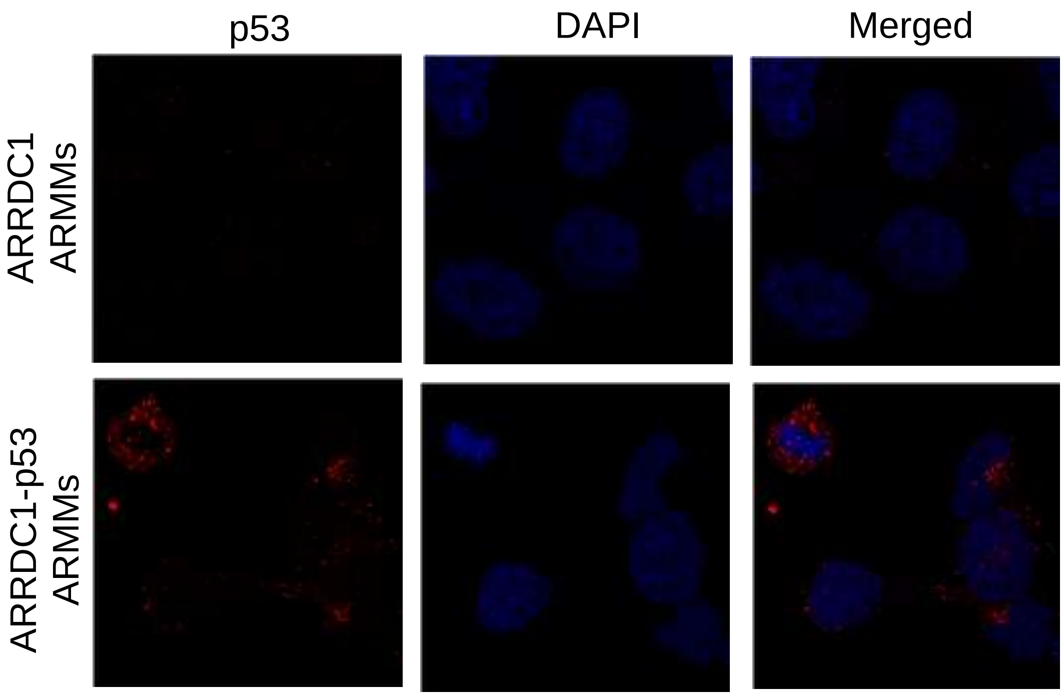

Supplementary Figure 4: Immunofluorescence staining of ARRDC1-p53 in recipient cells. H1299 cells were incubated with ARRDC1- or ARRDC1-p53 ARMMs for 16 h, washed with PBS extensively, and subjected to p53 immunofluorescence staining. DAPI was included to stain nuclei.

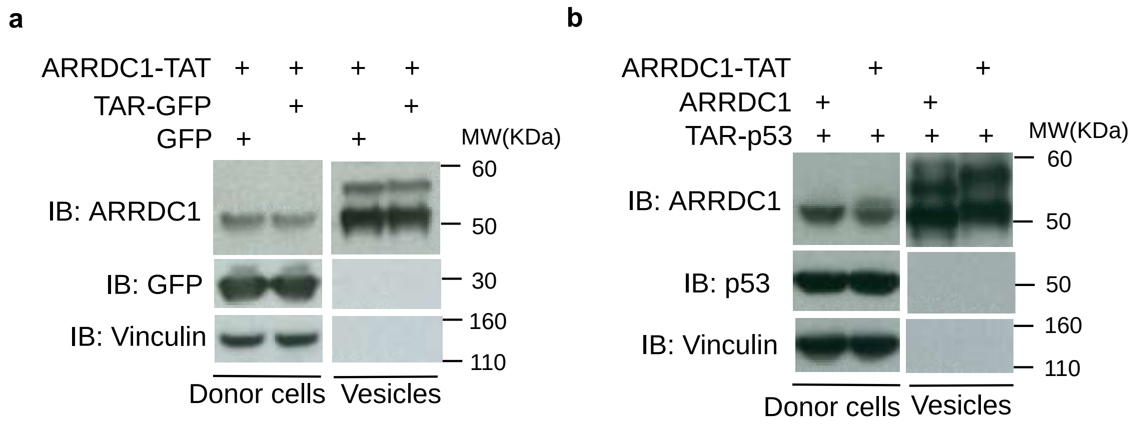

Supplementary Figure 5: No GFP or p53 proteins were detected by Western blot in either GFP or TAR-GFP-mRNA-containing ARMMs. (**a**) GFP or TAR-GFP was co-transfected with ARRDC1-TAT into the product cells 293T. (**b**) ARRDC1 or ARRDC1-TAT was co-transfected with TAR-p53 into HEK293T cells. Medium was collected for vesicle isolation. Cell lysates and vesicles were subjected to Western blot analysis using indicated antibodies.

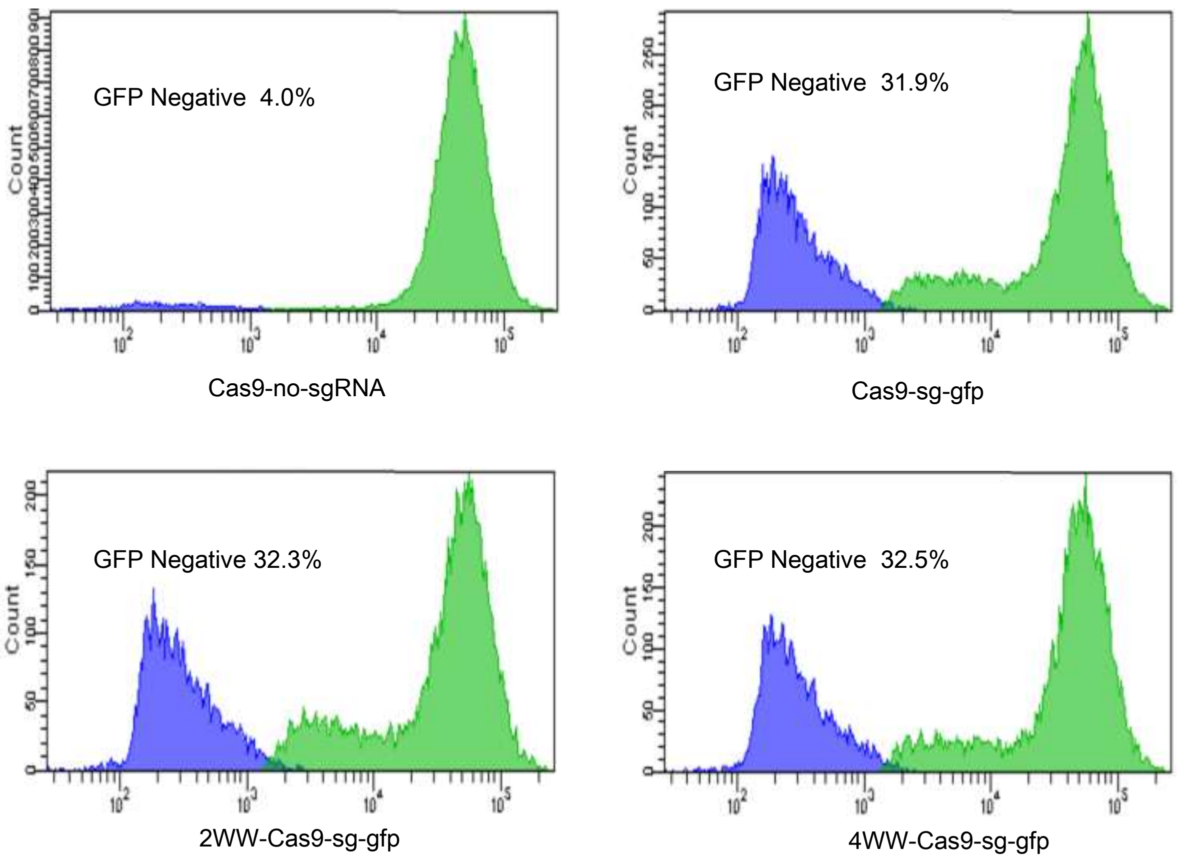

Supplementary Figure 6: Flow cytometry data showing gene editing activity of control and WW-linked Cas9. U2OS cells with stable integration of a single copy of GFP gene in the genome were transfected with Cas9 containing no guide RNA, Cas9 with GFP-targeting sgRNA, 2WW or 4WW-linked Cas9 with the sgRNA. 48 h post transfection, the cells were used in flow cytometry to assess GFP expression.

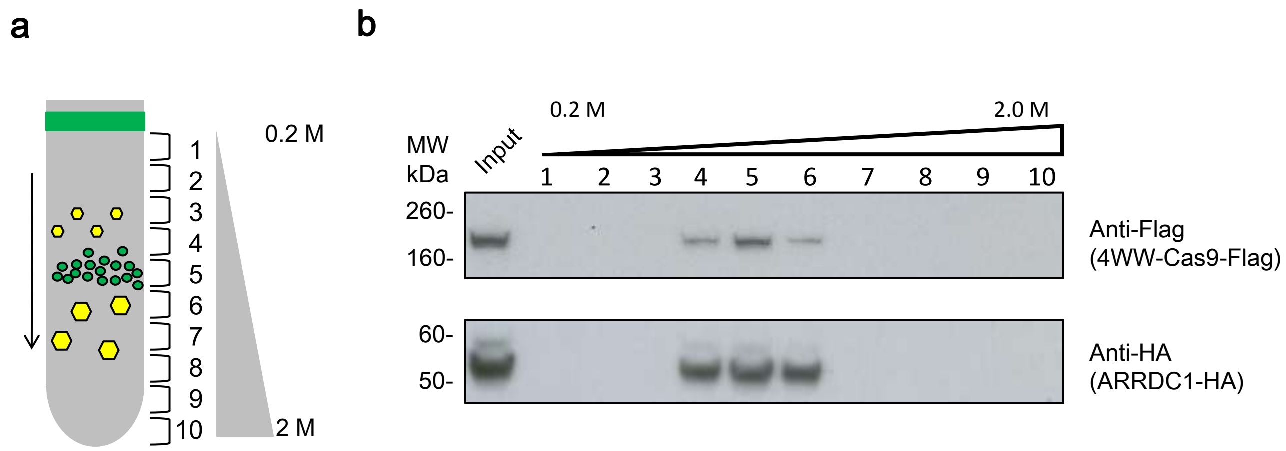

Supplementary Figure 7: Co-segregation of WW-Cas9 with ARMMs. (**a**) Schematic of the sucrose gradient fractionation of EV pellet via ultracentrifugation. EV pellet collected from HEK293T cells co-transfected with 4WW-Cas9 (Flag tagged) and ARRDC1-HA was loaded onto a sucrose gradient (0.2–2 M) and centrifuged at 180,000×*g* for 18 h. Fractions were then carefully collected at 1 ml each from the bottom of the tube. (**b**) Western blotting of fractionated samples. Each of the ten fractions from sucrose gradient ultracentrifugation was spun down by ultracentrifugation. Pellets were then used for anti-Flag (to detect 4WW-Cas9-Flag) and anti-HA (to detect ARRDC1-HA) Western blotting.

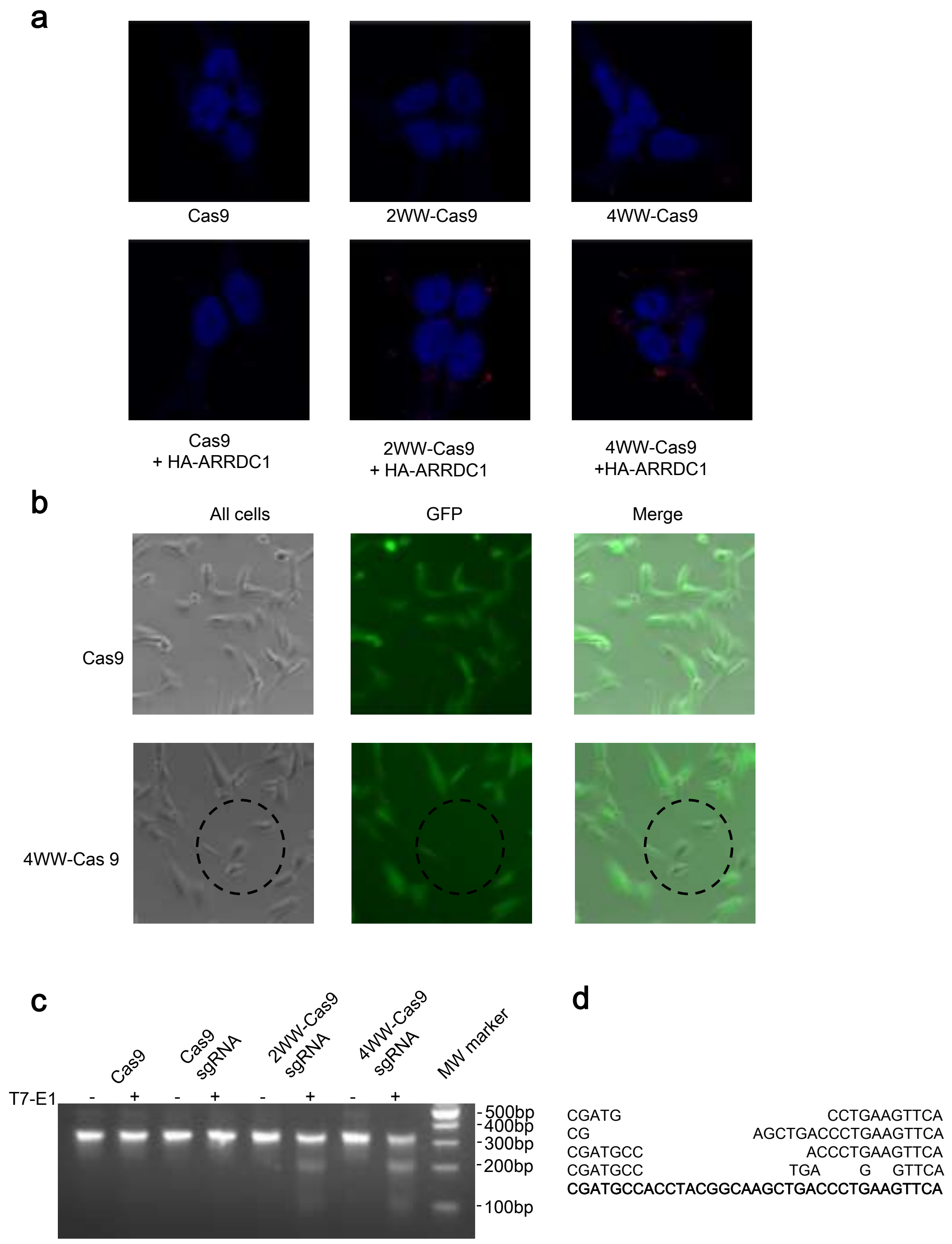

Supplementary Figure 8: (a) HEK293T cells were incubated for 24 hours with ARMMs collected from cells transfected with indicated constructs. The cells were then fixed and stained with anti-Flag antibody (to detect Cas9 which is fused to Flag tag) and a fluorescence-labeled secondary antibody. Confocal imaging was done using Leica SP8 X microscope. **(b)** U2OS cells with stable integration of a single copy of GFP gene were incubated with Cas9 ARMMs or 4WW-Cas9 ARMMs. 48 hours later, cells were imaged by Nikon Eclipse Ti-S microscope. **(c)** **(d)** GFP-U2OS cells were incubated with different ARMMs. 48 hours later, GFP-negative cells were sorted out. Genomic DNAs were extracted and used for PCR amplification of the GFP gene, followed by T7E1 assay **(c)** or TA cloning and direct sequencing **(d)**. Four out of 20 direct sequencing results showed indels around the sgRNA-targeting region **(d)**.

Supplementary Figure 9

The scans for all the original films.

For Fig-1-c and d

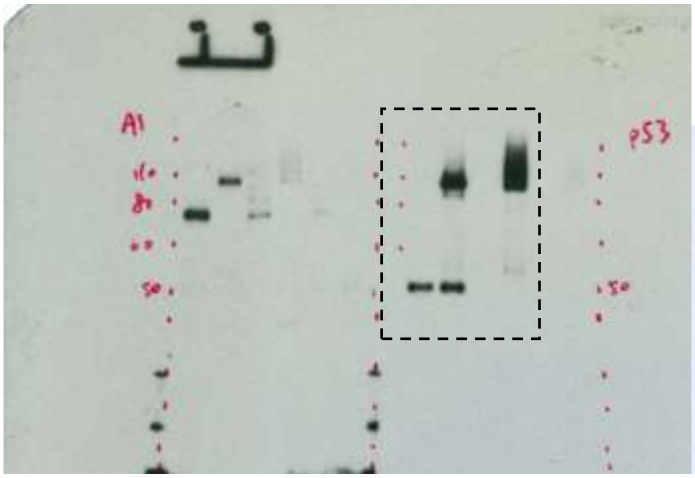

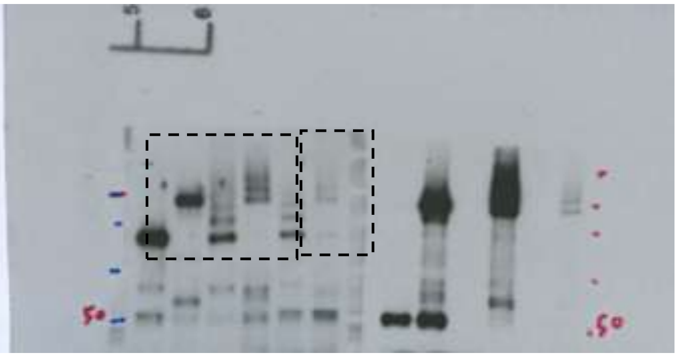

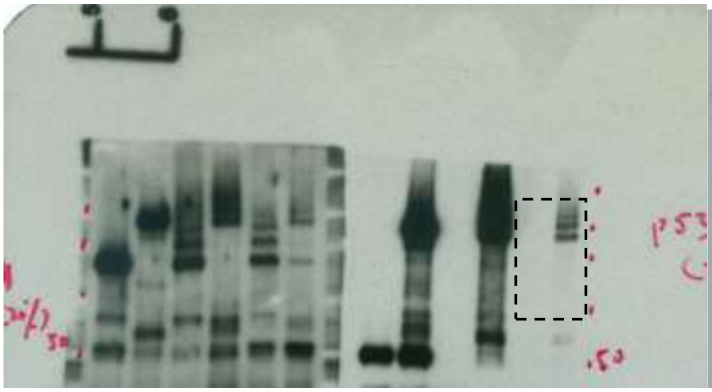

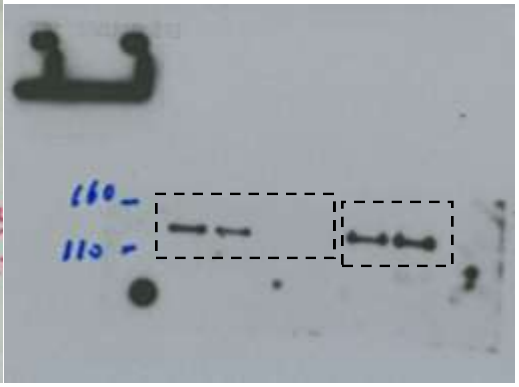

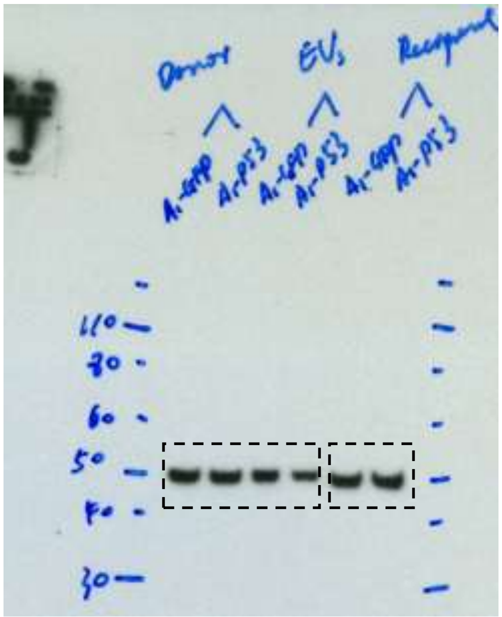

For Fig-3-b

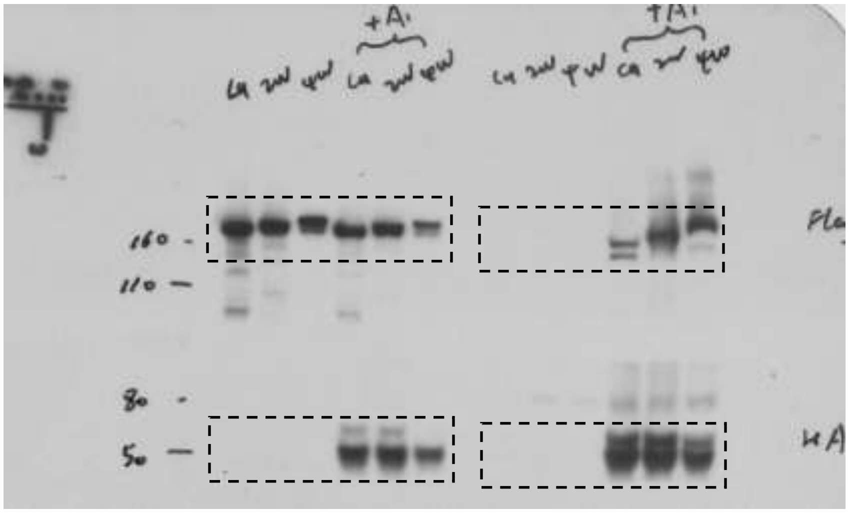

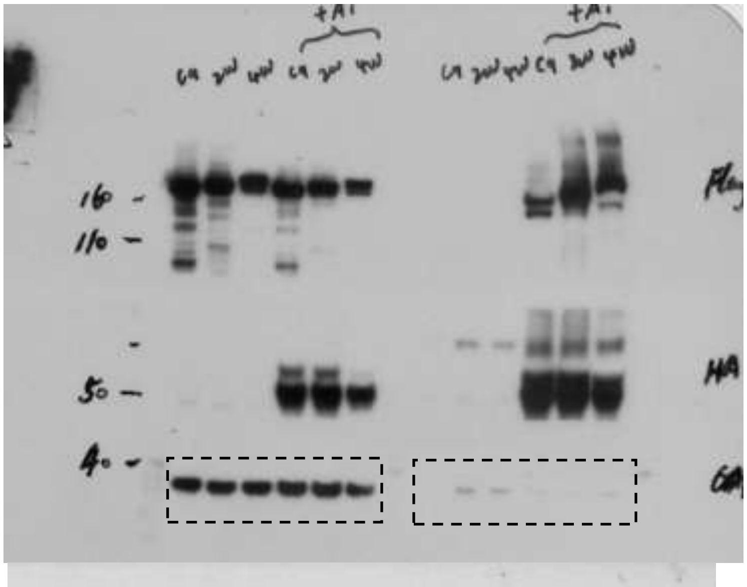

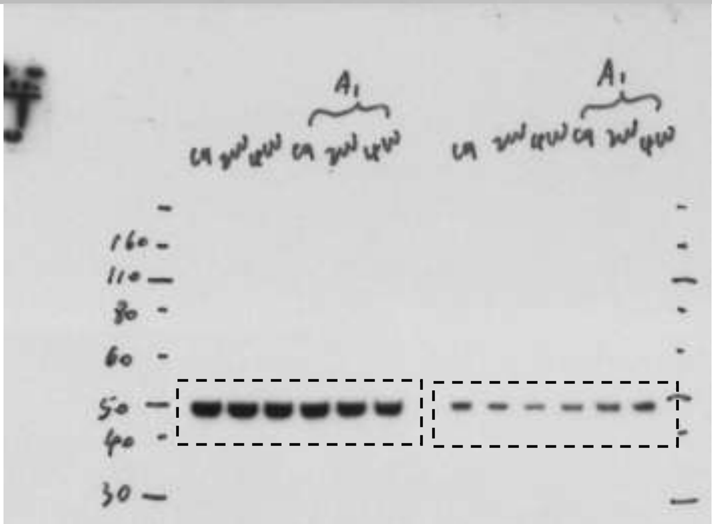

For Fig-3- d

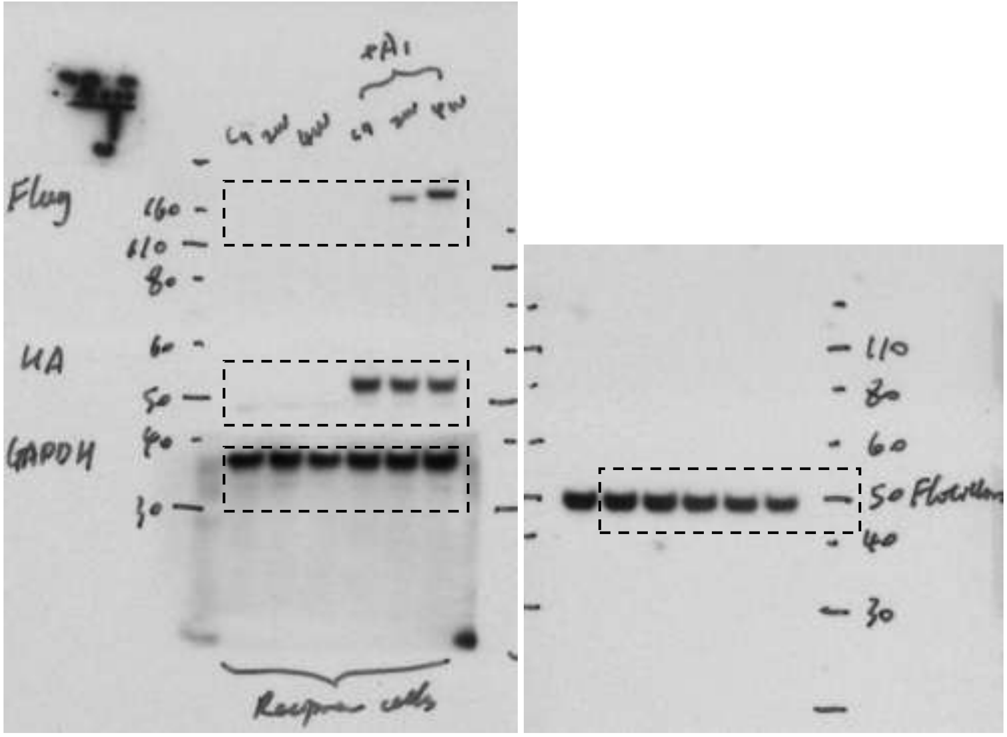

For Supplementary Figure 1-a

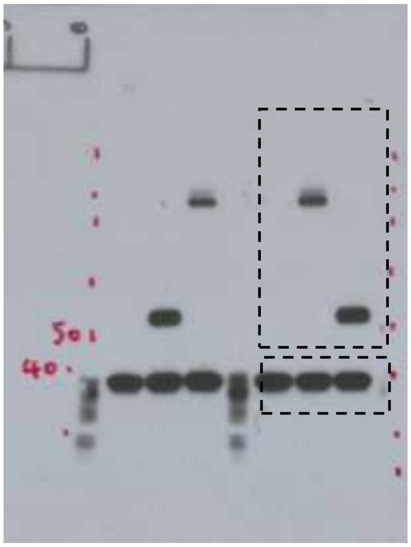

For Supplementary Figure 3-a

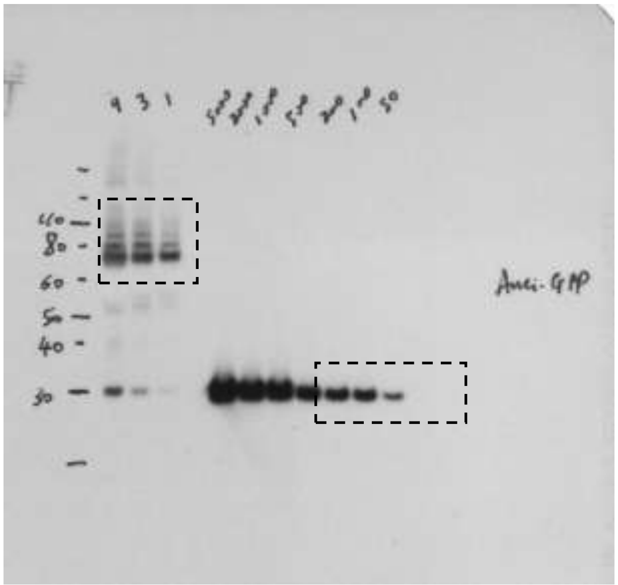

For Supplementary Figure 3-b

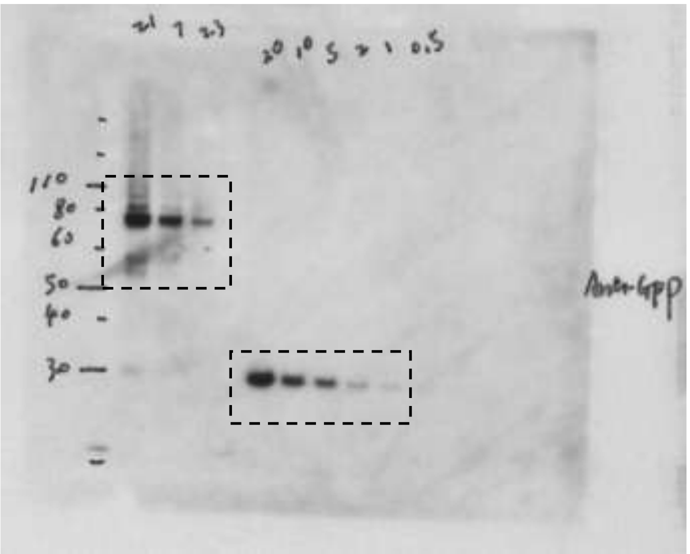

For Supplementary Figure 5-a

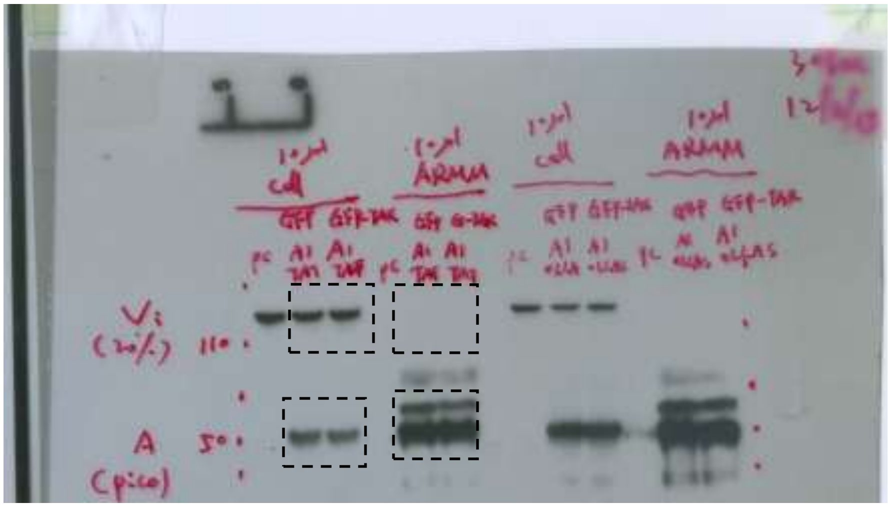

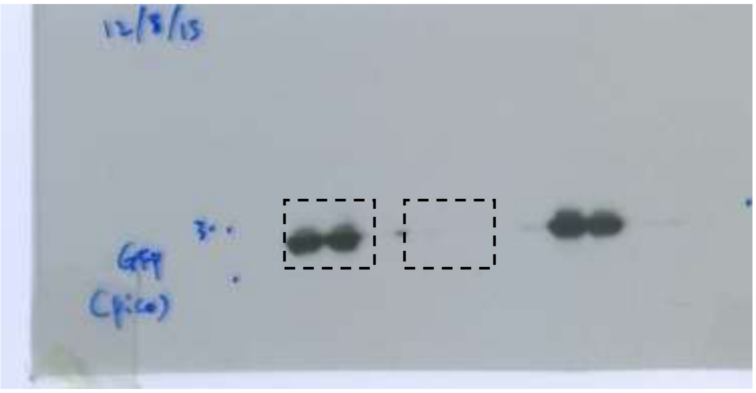

For Supplementary Figure 5-b

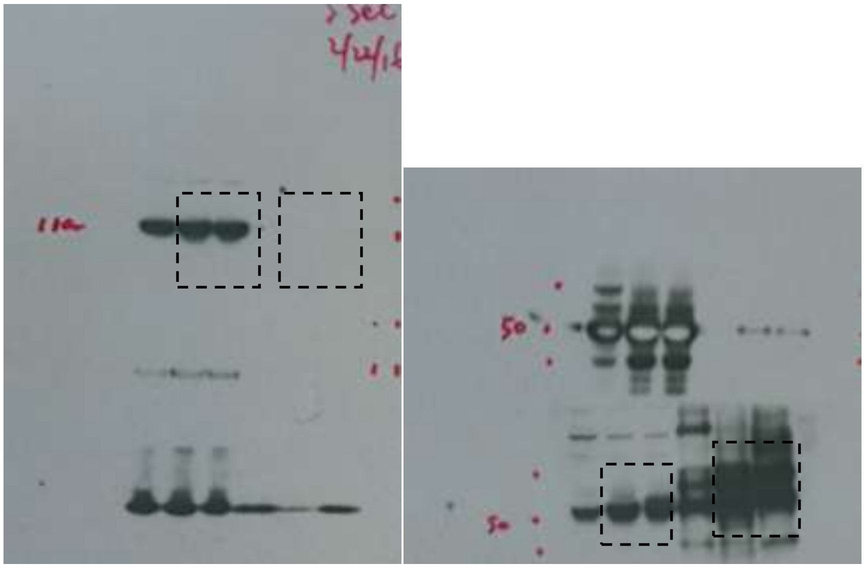

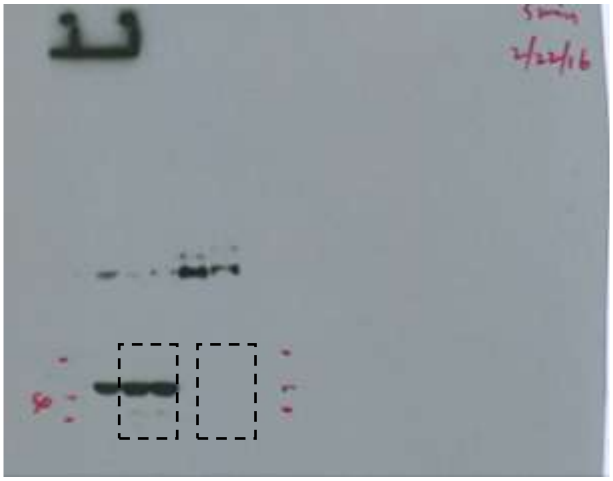

For Supplementary Figure 7-b

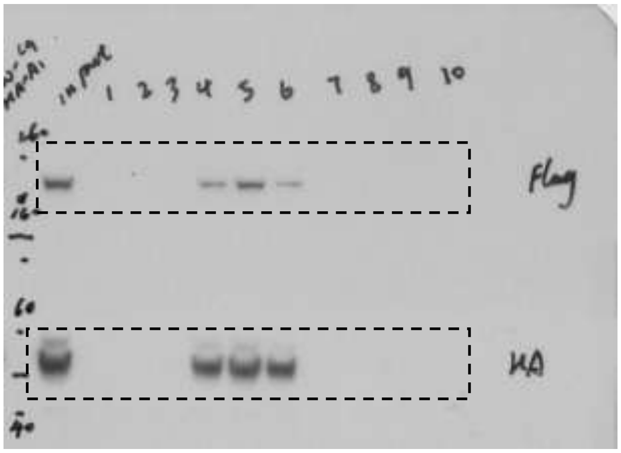

Supplement: Supplementary file 1 — Supplementary Information [file 41467_2018_3390_MOESM1_ESM.pdf]
